# Can someone look after my children while I write this COVID-19 paper?

**DOI:** 10.1007/s00259-021-05496-9

**Published:** 2021-09-01

**Authors:** Elske Quak, Gilles Girault, Charline Lasnon

**Affiliations:** 1 Comprehensive Cancer Centre F. Baclesse, Nuclear Medicine Department, UNICANCER, F-14000 Caen, France; 2 Comprehensive Cancer Centre F. Baclesse, Medical Library, UNICANCER, F-14000 Caen, France; 3 UNICAEN, INSERM 1086 ANTICIPE, Normandy University, F-14000 Caen, France

Dear Editor,

In 1949, French philosopher Simone de Beauvoir wrote the following in her book *The Second Sex*: *“Never forget that a political, economic or religious crisis will be enough to cast doubt on women's rights. These rights will never be vested. You'll have to stay vigilant your whole life.”* Unfortunately, this quote fits the current health crisis we are going through all too well.

It is in this context that we read the Editorials by Ekmekcioglu et al. [1] and Gelardi et al. [2] on female scientific and academic careers in nuclear medicine and the EANM Women Empowerment initiative (EANM WE). Unsurprisingly, Gelardi et al. found a male overrepresentation on the Editorial boards of the *EJNMMI* Journal Family as well as gender-related differences of the H-index in favor of men.

In general, the literature on gender gaps in science consistently shows the underrepresentation of women. The higher up the career ladder the worse it gets, despite the growing numbers of women in the workforce. Gender bias and discrimination, the motherhood penalty, sexism, male-dominated cultures and networks and the unbalanced division of parental and domestic tasks [3] are some examples of challenges women face.

To rub salt in the wound, the COVID-19 pandemic has tended to aggravate existing social inequalities. A survey among principal investigators in science by Myers et al. showed the unequal effects the pandemic has had on scientists, with female scientists with young children being disproportionally affected [4]. For those who recognize themselves as part of this disadvantaged group, many of us will probably have realized that our ability to work during lockdowns mainly depends on schools and kindergartens staying open. By temporarily diminishing scientific productivity, the pandemic might thus have long-lasting effects on careers.

On the other hand, the pandemic has offered, and is still offering, unique opportunities for research. COVID-19-related publications are flourishing, in nuclear medicine too. From recent data, we know that women represent one in three first and one in five last authors in medical imaging [5, 6]. However, will these proportions be the same for COVID-19-related papers in nuclear medicine, necessarily written during the pandemic, or will we find an underrepresentation of female authors?

To find out, we conducted a bibliometric analysis of first and last author gender of publications retrieved on PubMed by using the following equation: ("Radionuclide Imaging"[Mesh] OR "Nuclear Medicine Department, Hospital"[Mesh] OR "Nuclear Medicine"[Mesh] OR "positron emission tomography" OR "PET/CT" OR "single photon emission computed tomography" OR SPECT OR scintigraphy) AND ("COVID-19"[Mesh] OR "SARS-CoV-2" [Mesh] OR "covid-19" OR "coronavirus disease-19" OR "coronavirus disease 2019" OR "SARS coronavirus 2" OR "SARS-CoV-2" OR "SARS-CoV2" OR "2019-ncov" OR "2019 novel coronavirus" OR "severe acute respiratory syndrome coronavirus 2"). Date of censoring was June 30^th^. Author gender was determined as per the previous described methodology [5]. Of the 377 PubMed references found, 326 (86.5%) presented complete datasets for first and last authors. We found that 36.5% (119 of 326) of first and 19.0% (62 of 326) last authors were female (Fig. 1). We did not have demographic data regarding age and parenting, so no correlations could be established. However, we can draw the preliminary conclusion that the gender gap in authorship of COVID-19-related papers in nuclear medicine did not widen. Future gender studies covering the entire duration of the COVID-19 pandemic will ultimately show whether it will have had an impact on female scientific output in general, including grant applications and the initiation/finalization of research projects during the pandemic.

**Fig. 1:**
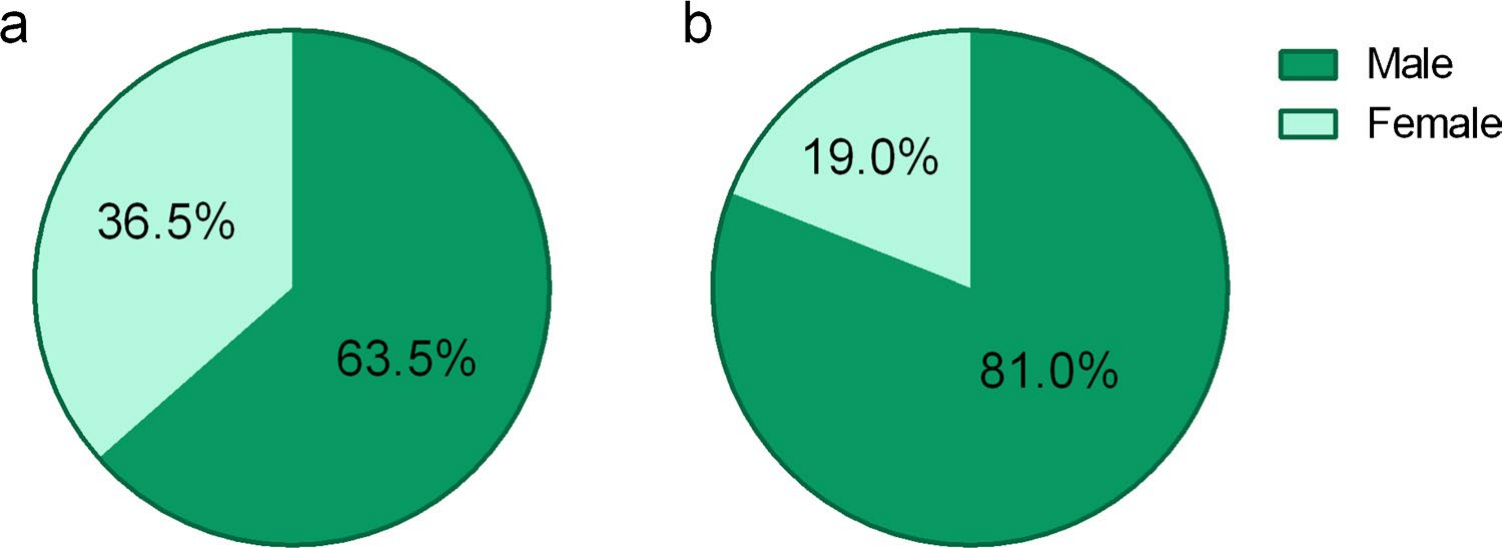
Pie charts showing gender distribution of first (**a**) and last (**b**) authorship of COVID-19-related papers in nuclear medicine

The Editorials by Ekmekcioglu and Gelardi and colleagues open the debate on female careers in nuclear medicine. We would like to add that the disruption caused by the pandemic could be the opportunity to accelerate the rate of change towards equity, diversity and inclusion. Needless to say, there is no easy solution for gender inequality. Gender diversity and inclusion are complex issues, depending not only on factors related to work, but also on history, culture and what society expects and accepts from women. Leaders and physicians should be aware of explicit and implicit gender biases and be educated about the challenges women and minorities face to climb the career ladder. When it comes to Women Empowerment, the term empowerment should not be mistaken for one of the myths about diversity and inclusion, namely “we have to fix the women” [7]. Teaching women to adopt male behavior to succeed will only lead to backlash and unhappiness. Instead, female leadership should become fully accepted. The path to equity demands a multifactorial approach [8] and perhaps a remodeling of parts of the system historically designed by men, in order to better fit both sexes. We are convinced that progress towards equity can only be obtained by a close collaboration between men and women of all generations.

## Data Availability

The dataset analyzed in the current report was the result of a free PubMed search and the use of the Gender-API software. The dataset is available from the corresponding author on reasonable request and after signature of a data access agreement.
